# Detecting and Responding to a Dengue Outbreak: Evaluation of Existing Strategies in Country Outbreak Response Planning

**DOI:** 10.1155/2013/756832

**Published:** 2013-10-09

**Authors:** Julia Harrington, Axel Kroeger, Silvia Runge-Ranzinger, Tim O'Dempsey

**Affiliations:** ^1^Liverpool School of Tropical Medicine, Liverpool L3 5QA, UK; ^2^Special Programme for Research and Training in Tropical Diseases (TDR), WHO, 1211 Geneva 27, Switzerland

## Abstract

*Background.* Dengue outbreaks are occurring with increasing frequency and intensity. Evidence-based epidemic preparedness and effective response are now a matter of urgency. Therefore, we have analysed national and municipal dengue outbreak response plans. *Methods.* Thirteen country plans from Asia, Latin America and Australia, and one international plan were obtained from the World Health Organization. The information was transferred to a data analysis matrix where information was extracted according to predefined and emerging themes and analysed for scope, inconsistencies, omissions, and usefulness. *Findings.* Outbreak response planning currently has a considerable number of flaws. Outbreak governance was weak with a lack of clarity of stakeholder roles. Late timing of responses due to poor surveillance, a lack of combining routine data with additional alerts, and lack of triggers for initiating the response weakened the functionality of plans. Frequently an outbreak was not defined, and early response mechanisms based on alert signals were neglected. There was a distinct lack of consideration of contextual influences which can affect how an outbreak detection and response is managed. *Conclusion.* A model contingency plan for dengue outbreak prediction, detection, and response may help national disease control authorities to develop their own more detailed and functional context specific plans.

## 1. Introduction

Dengue, a mosquito-borne viral disease, is emerging as one of the world's most rapidly spreading and important infectious diseases of the twenty first century [1] A somewhat different disease scenario exists today, with all four viral serotypes circulating in Asia, Africa, and the Americas, an estimated 3.6 billion people living in dengue endemic countries, and over 50 million dengue infections occurring annually [2]. The increasing global threat of dengue outbreaks in both endemic and nonendemic regions has led to a focus on establishing an effective outbreak response. A dengue outbreak response has been defined as the sum of measures specifically addressing a dengue outbreak, with the aim of reducing case fatality rates, case number, and entomological parameters [3]. In addition to this, in order to detect the outbreak, systematic surveillance needs to be in place.

Emergency preparedness and anticipated response planning are an integral part of dengue control, yet this is often neglected in dengue endemic countries [1]. Different measures need to be implemented depending on the context of dengue in the area. Thus, in endemic areas, the ability to identify and coordinate an outbreak response should be a priority, whereas in dengue free areas, strategies are based on reacting to sporadic cases, risk indicators, or alert signals. Some principles of outbreak response planning are highlighted in the following.

*Components of Emergency Outbreak Response Planning*:

- clearly articulated aims, objectives, and scope,
- a lead coordinating agency,
- establishing an emergency response plan,
- organisational links with other agencies that have direct responsibilities for implementation of the plan,
- specific roles and responsibilities of key agencies documented,
- costs and resources highlighted,
- distribution of the plan to all response and supporting agencies,
- monitoring and evaluation framework,
- clear triggers for the activation and deactivation of the plan,
- objective criteria for defining an outbreak based on specific local data,
- multisectoral exercises to discuss and validate the plan,
- formal debriefing sessions to partners.

Despite the wide variety of different interventions in place, there is the scant literature evaluating outbreak response activities and there are no evidence-based universal strategies for management of dengue epidemics that can be used as the basis for the development of context specific outbreak response plans. Studies evaluating outbreak responses have been difficult to interpret as they generally describe a wide range of interventions implemented in different ways, and the available literature focuses largely on epidemiological surveillance or vector control [3]. The lack of evidence-based outbreak response strategies led to a global research agenda set up by the Scientific Working Group at WHO in 2006 recommending case studies of national programmes to identify factors leading to success or failure of dengue prevention and control programmes [4]. It has also been recommended that future research should compare national and international policies in emergency response plans to identify the common interventions currently described [3]. The aim of this research was to contribute to an improved response to dengue outbreaks through the comparison and analysis of existing strategies and dengue outbreak response plans.

## 2. Methods

### 2.1. Data Collection

This research is part of a programme funded by the European Commission and coordinated by the TDR-WHO (Special Programme for Research and Training in Tropical Diseases), intending to identify strategies in dengue outbreak detection and control in order to improve future responses. The comparison of country outbreak response plans involved the acquisition of grey literature from governments and organisations which had existing outbreak response plans. The literature was obtained through the World Health Organisation (Department of Neglected Tropical Diseases). The inclusion criteria consisted of any regional, national, provincial, or local dengue policy that contained details on outbreak response strategies, from any source, in English, Spanish, and Portuguese. Three of the documents, originally in Spanish/Portuguese, were translated into English for this study. A systematic screening of material allowed the exclusion of incomplete documents, documents with no details of outbreak activities, and draft strategies comprising of only recommendations for a dengue outbreak response. Limitations of the methodology included the following: the sampling of plans was largely out of the researchers' control in that they were provided by the WHO; there may be a number of functioning plans that were not obtained, particularly in other national/local languages; and the English translation of Spanish/Portuguese texts may have led to misinterpretation of the information; thus, some information may have been missed or lost. Due to the diversity of plans to be compared, collectively obtaining information under headings was sometimes difficult, and occasionally, inferences had to be made to ensure comparability. 

### 2.2. Analysis

The country dengue outbreak plans were analysed using a data matrix. A framework approach was used, whereby information was extracted using predefined themes, but additionally emerging themes were added so that if one plan revealed additional information about a pertinent component of the outbreak, it could be added to the data codes and previously read plans scrutinized to confirm the absence of this information [5]. A critical analysis of the grey literature was the method of analysis, whereby plan structure and content were appraised for clarity, detail, applicability, instructional capacity, and usefulness to public health providers. International recommendations were also taken into account [6]. 

## 3. Results

Fourteen documents were obtained containing details of the activities implemented in relation to a dengue outbreak. Due to the relative paucity of specific outbreak response plans, the documents reviewed were heterogeneous in their approach. Of the fourteen plans, nine were outbreak response plans; however, two of these were guides for all infectious disease outbreaks that included an annex on dengue, leaving seven dengue specific outbreak control plans. Of the others, four plans documented control of dengue in the interepidemic period, with some outbreak response measures included. One of the plans was a “best practice” set of recommendations for control of dengue.

### 3.1. Outbreak Management and Stakeholders

Outbreak planning must ensure that governance over the response is present, particularly through highlighting the stakeholders to be involved, providing details of monitoring of the response, ensuring that transparent risk communication is in place, and allowing for human resource preparedness planning. Of the fourteen plans, ten included a section on the key stakeholders involved in dengue control, with an additional two informally highlighting stakeholder roles. There was great diversity in the stakeholders involved in the outbreak response, often depending on service providers in each country. Documented sectors the plans stated to be involved included entomologists (4/14), the environment sector (6/14), non-governmental organisations (5/14), private healthcare facilities and additional private institutions (5/14), civil societies (8/14), and the education sector (8/14). Despite all of the plans stating the importance of vector control in an outbreak, the majority failed to document that entomologists or vector control stakeholders should be involved. Only six of the fourteen plans contained a formal section on the intersectoral approach in the dengue outbreak response. Of the fourteen plans, ten recommended the establishment of a specific team to control a dengue outbreak, with eight of the ten plans documenting specifically who should be in this team. The team members varied depending on the key stakeholders that already functioned in healthcare delivery, disaster control, and interepidemic dengue prevention. However, some core members were recommended by a number of plans: clinicians/nurses (5/14), laboratory representatives (5/14), public health staff (5/14), local government or leaders (4/14), vector biologists (3/14), and environmental representatives (3/14). Despite this, the documentation of an intersectoral approach to a dengue outbreak was weak in relation to functionality. The information provided on the roles and responsibilities of the stakeholders was often minimal for an outbreak, with some plans simply stating their presence. Some plans took a very top-down approach, whereby the ministries to be involved were documented; others took a bottom up approach, recommending local stakeholders. 

Of the fourteen plans, thirteen reported monitoring and/or evaluating a dengue outbreak response, which involved monitoring of cases throughout the outbreak (8/14), and monitoring entomological indices (6/14). There was often a lack of clarity as to how monitoring and evaluation should be conducted during an outbreak, with few plans recommending a combination of epidemiological data and entomological indicators to ensure successful control (2/14). Risk communication is a prominent part of an outbreak response, and many documents stated the need to regularly update the public on the outbreak status (9/14), focusing on communication through the media (8/14). There was little documentation of specific risk communication to clinicians and healthcare workers (2/14). Documentation of how, when, and through whom risk communication should occur was weak. Ten plans acknowledged the need for human resource preparedness for a dengue outbreak. Recommended strategies included staff training during the interepidemic period (9/14) emergency training to be provided during an epidemic (2/14), and staff recruitment from other areas (6/14). In the majority of the plans, there was a lack of detail specifying who should be trained or recruited, or how this training would take place, by whom, and how often. Other strategies advocated in individual plans included maintaining an emergency staff roster, maintaining a list of unemployed nurses and healthcare assistants, recruitment from civil societies and the private sector, and mobilisation from the Emergency Operations Committee.

### 3.2. Surveillance

Twelve of the plans included information about dengue surveillance. All twelve predominantly documented the use of a passive surveillance system to detect an outbreak, reliant on reporting from healthcare professionals to a local or central unit (12/14). One of the main functions of an effective surveillance system is to highlight early an increase in case number in order to identify an epidemic and initiate prompt action. The passive system is dependent on case reporting by clinicians, yet half of the plans did not state that it is necessary for *all* health units to report (7/14). Only half of the plans stated how reporting would take place (7/14), with a significant lack of electronic systems (2/14). Just under half of the plans did not state *who* should be notified on suspicion or confirmation of a dengue case (6/14). The process of analysis of dengue case data (particularly transfer from a local to a central level) was included in a few plans (5/14), with only one plan stating that analysis would occur at the local level. 

Enhanced surveillance methods were either documented for the interepidemic period, or to be introduced when an outbreak is suspected or confirmed. Collectively, enhanced surveillance strategies were sporadically documented in the plans. Active surveillance was the most commonly documented strategy to be introduced (7/14), with some plans specifically recommending sentinel surveillance (4/14). Other approaches included syndromic surveillance (2/14), mortality surveillance (2/14), and severe case surveillance (2/14). Laboratory support was documented to be a key part of the confirmation of dengue cases (either confirming a proportion or all of cases) in twelve of the fourteen plans. However, only a few plans documented exactly what tests should be performed (9/14), at what exact stage in the illness (5/14), and the number of tests required to substantiate an outbreak when resources are limited (7/14). Logistical considerations were only occasionally taken into account (5/14). Isolating the serotype and referral to a reference laboratory for quality assurance was also documented (6/14 and 9/14, resp.). 

### 3.3. Outbreak Definition, Alerts, and Verification

In order for an outbreak response to be initiated in the first instance, an outbreak should be clearly defined. Of the eight plans that document a definition, there was use of both case numbers (4/14) and surveillance thresholds (4/14) to define the outbreak. In addition, in order to react early to an outbreak, a few plans recommend alert triggers that may inform a health system or government to the threat (8/14). Epidemiological (5/14), entomological (2/14), laboratory (2/14), and geographical alerts (1/14) were recommended. Some plans document parameters that should trigger enhancement of preventative or routine measures, in a so-called alert phase. No plan stated the use of climate data to predict an outbreak. Of the plans that stated alerts for an outbreak, some documented actions to be taken, such as more data collection or the introduction of active surveillance, but this was still missing in some plans which compromised the meaning of alerts stated (5/14). 

 An outbreak investigation was recommended to take place by most of the plans (10/14), yet the process varied significantly, dependent on endemicity levels, previous experience with outbreaks, and resources available. Common elements of recommended outbreak investigations were obtaining a travel history of the index case (6/14), filling out the case report form (6/14), conducting vector surveillance (8/14), and active case finding (8/14), all of which aim to confirm the case and the outbreak. There was poor documentation of the stakeholders responsible for investigating the outbreak (5/14), and less than half of the plans advocated a risk assessment for dengue outbreaks to be performed (5/14). 

### 3.4. Outbreak Response

All plans documented vector control measures that should be implemented in an outbreak; all stated a role for larval control in the form of source reduction efforts (14/14), the majority stating a role for chemical control (9/14), and a minority for biological control (5/14). Ten of the plans advocated adult mosquito control, predominantly through chemical methods involving the space spraying of insecticide (8/14 outdoor spraying, 3/14 indoor spraying). Nonchemical mosquito control methods were rarely documented (2/14). The major gaps in vector-control documentation included who is responsible for such actions, when and for how long vector control should be performed, mapping of the area that needs to be covered around the case residence, and monitoring of the vector population. The plans often focused on environmental larval control during the interepidemic period but emphasised chemical mosquito control during the outbreak, usually in the form of space spraying. The more robust plans stated context specific details of how larval control would be deployed. The community (4/14), government teams (5/14), and NGOs (2/14) were all mentioned in different plans as being responsible for source reduction.

All plans recommended some form of community engagement during a dengue outbreak, with the focus on community education (13/14). A minority of the plans focused on direct mobilisation of the community in order to either spread educational messages to or promote destruction and prevention of mosquito breeding sites (6/14). Some good practices have been identified from the plans. At a governmental level, this includes targeting specific human resources to help in an outbreak, organising community participation schemes, and coordinating mass media campaigns. At the level of the community, particular actions include meetings with leaders or social representatives, community mobilisation via advertising, and making individuals aware of the responsibility they possess to aid in outbreak activities. Three types of educational message have been identified: (a) preventative measures that focus on the interruption of dengue mosquito breeding sites, (b) protective measures to prevent exposure to mosquitoes, and (c) encouragement of health seeking behaviour. Rarely did a plan include all three of the messages to be communicated to the public.

Health service management is a crucial consideration when contingency planning, yet this was relatively neglected in the plans, with only half of the plans documenting how the structure of health services should be considered in an outbreak (7/14). An outline of how a health service would adapt to an influx of patients was sporadically documented in only the more robust plans. Some strategies documented included the implementation of an effective triage system (4/14), the establishment of an emergency room (2/14), the identification of additional facilities to be used (3/14), preparation and mobilisation of resources (6/14), and transmission control in hospitals (7/14).

## 4. Discussion

Outbreak response planning has been identified as a way to augment engagement of partners, build capacity, and develop infrastructure, providing operational links to ensure a structured and coordinated response [7]. The current thinking in outbreak response planning in public health recommends that outbreak planning needs to be locally adapted depending on the presence or absence of public health infrastructure for each individual disease [8]. In this review, just one of the 14 plans provided an holistic and comprehensive picture of how the surveillance system and response plan should be organised in order to (a) detect a dengue outbreak at an early stage through clearly defined and validated alert signals, (b) exactly define that a dengue outbreak has started, and (c) organise an early response to the warning signals detected, or a late response when an outbreak has been verified [9]. By combining the information from the different plans analysed in this paper and adding information from WHO documents, a clearer picture emerges about the essential elements of a comprehensive outbreak response plan. 

### 4.1. Outbreak Management

Control activities for a dengue outbreak need to be multisectoral, multidisciplinary, and multilevel, requiring environmental, political, social, and medical inputs to be coordinated so that productive activities of one sector are not negated by the lack of commitment from another [3]. This is required at all levels of surveillance, transmission control, and clinical management. Documenting the relevant stakeholders who should be involved in an outbreak response, both at a local level with regard to implementation, and at a higher political level with regard to decision-making, is crucial in order to coordinate the response. Yet, there is often a neglect of documentation of stakeholder roles in outbreak response plans, leading to a failure to acknowledge the importance of intersectoral communication, a failure to recognise capacity, a lack of appropriate delegation, and a lack of accountability with regard to activities. The literature highlights how the coordination of a diverse set of sectors and social groups has faced challenges. Often mechanisms do not exist to guarantee intersectoral compliance with a regulatory framework in terms of financial and operational participation, thus weakening the outbreak response, due to delegation to stakeholders with poor operational organisation [10]. Recognition of where capacity lies is thus far more important than encouraging universal sectors to be involved in an outbreak response. Nevertheless, this review has found even sectors that do have capacity are often not documented to be involved in managing an outbreak response. Current planning focuses on “what” should be implemented, without considering “who” shall be responsible, with a distinct lack of functional detail of the roles of stakeholders. 

 Acknowledgment of the additional human resources that will be required in a dengue outbreak, both in clinical management of cases and in transmission control, is relatively well covered in outbreak response planning. Redistribution of staff and upscaling of roles of existing staff is one method of ensuring adequate staff numbers, whereby medical personnel may be required to work longer hours and move to other locations [11]. Yet, the overworking and consequent demotivation of public health staff may be a barrier in a dengue outbreak response, and plans may fail to take into consideration the difficulties facing redistribution of relevant trained professionals in practice [12]. The benefit of staff training for an outbreak in the interepidemic period has been established in the literature and in this plan analysis, and experience has led to a recognition of the additional use of hands-on training during a dengue outbreak [3, 13, 14]. Thus, investment in human resources must come prior to the outbreak. This may be difficult for countries that have weak governments. Outbreak response planning documentation should include a section specifying activities that should be performed in the interepidemic period in preparation for an outbreak, particularly related to outbreak preparedness as opposed to just preventative control. 

### 4.2. Surveillance

In an early literature review of epidemiological dengue surveillance, it was found that despite 46 of the 56 major dengue endemic countries in the world having functioning dengue surveillance systems, only four of these were robust enough for epidemic prediction [15, 16]. The literature highlights that the weaknesses of surveillance in predicting an outbreak continue to compromise the efforts of health systems to identify an outbreak in a timely manner. The initiation of this process has proved to be weak, with the underreporting of cases undermining the ability of surveillance to substantiate an outbreak and gaps in reporting being often identified, for example, whereby some surveillance systems only report dengue cases in children [17, 18]. The plans predominantly highlighted a dependence on weak disjointed passive surveillance systems. Indeed, there is often fragmented reporting, inadequate methods of notification, and weakness in the transfer of case information from local to central level for analysis, with a lack of local use of analysis data. This may lead to neglect of an immediate response, especially if a number of intermediaries are in place as has been identified [19]. It may be suggested that although a coordinated response from local government may be difficult in some settings, the use of local data for risk communication to the public, clear reporting flowcharts in place, a functional feedback system, data analysis also at the lowest possible level, and capacity building in surveillance at the periphery may be crucial. A lack of accountability at a local level needs to be challenged in order to successfully shorten the time delay between the onset of and response to an epidemic. A functional national surveillance system is paramount in outbreak response and must be incorporated into planning in order to target all the above weaknesses that currently exist. There is also a need for a more effective strategy in enhanced surveillance taking into account seasonal trends, something which is currently neglected in relation to outbreak detection. 

Laboratory surveillance needs to be strengthened for outbreak preparedness and response, and all but two of the plans stated a need for laboratory facilities, with quality control being crucial. Yet it has been found that many countries do not have the facilities in place to process such tests in a timeframe that is conducive to case confirmation for public health intervention [20]. If countries do not have such facilities in place, working with clinical case definitions may need more prominence in outbreak response planning. In particular, the value of clinical identification of warning signs for severe dengue, and knowing how to triage patients in the absence of laboratory test results may be crucial. It may be argued that unless there are details of which tests need to be performed at which point in the clinical illness, recommending laboratory testing may actually impede good clinical management and identification of an outbreak.

### 4.3. Outbreak Definition, Alerts, and Verification

A first and fundamental gap in outbreak response planning is the lack of clarity offered when deciding what an “outbreak” actually is. A significant proportion of the plans analysed does not provide an outbreak definition. Therefore countries are working with plans that do not explicitly state when their plan should be implemented. Outbreak definitions that fail to relate cases in time and place may cause problems for dengue endemic countries because a certain number of cases will be occurring all the time and will usually increase during the rainy season. There is a requirement for laboratory confirmation in some of the outbreak definitions which may increase the delay in outbreak verification. The advantage of laboratory testing is that it allows diagnosis to be more specific, which is especially of use in highly endemic settings [21]. Terminology used, such as a “cluster of cases” may be ambiguous and lead to discrepancies in what stakeholders envisage as an outbreak. Often outbreak definitions rely on the timely analysis of surveillance data to establish that cases are above a threshold, yet surveillance systems are often incomplete, inaccurate, and slow, which undermines this process. 

**Figure 1:**
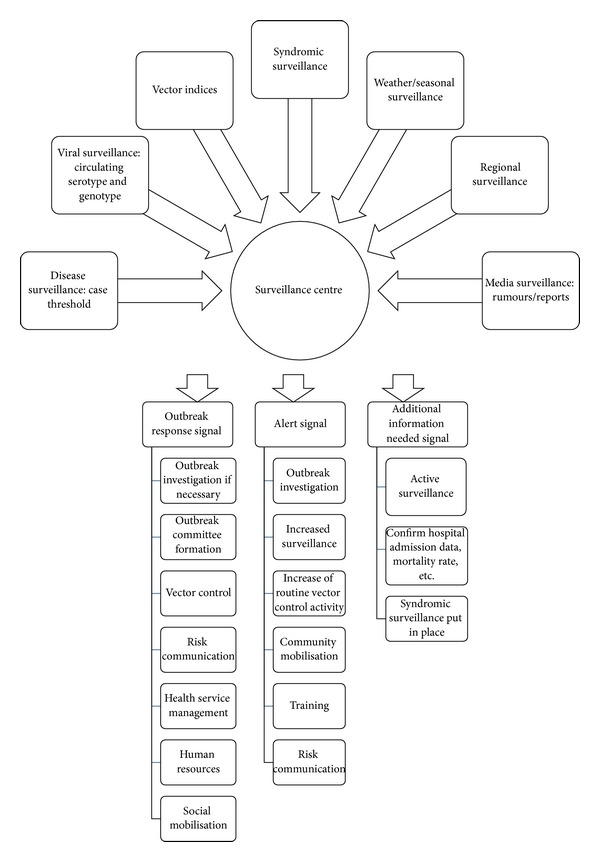
Alert signals of a dengue outbreak and possible Interventions.

Additional outbreak alerts, besides passive surveillance, have been proposed to be of great importance in areas where disease surveillance may be weak, or dengue transmission is endemic [22, 23]. Alerts are documented in a number of the plans, yet no single marker other than dengue case incidence stands out as an outbreak alert. Epidemiological parameters such as an increase in deaths, an increase in proportion of negative malarial cases among febrile patients, and an increase in hospital admissions may be easy to identify if routine data is collected. Alert signals based on laboratory information may provide a rapid alert if facilities are available. However, with regard to entomological surveillance alerts, questions have been generated concerning its effectiveness in predicting outbreaks particularly because the thresholds are unclear and may be highly dependent on local factors [24]. Few plans document that a rising case number should be considered as an early warning indicator, unless the number exceeds the threshold for a full outbreak response. This “crisis mentality” creates a dichotomous system whereby alerts split dengue activity into “outbreak” and “no outbreak” without any circumstances in between [15, 16]. This raises the question whether there is an opportunity for alerts to be based on a scale, so that if case number rises above a pre-defined alert threshold, some action can be taken which may increase capacity for outbreak control (i.e., an early response or initial response). It may be argued that a false alert due to low specificity of an alert signal may not waste resources if the correct public health interventions are implemented and preparedness measures taken, and this may be a way of utilising additional signals without deploying a full outbreak response [21]. An example of a tool to use is given in Figure 1. The alert tool will be characterized by thresholds based on the country's surveillance system taking into account seasonal trends, and in combination with other signals, the country deems appropriate, and contextualized depending on a country's resources. It is suggested that this tool needs to focus on the formation of readily identifiable triggers for a given country, be specific, and include a link to specific activities to be coordinated at the point of alert identification. This may aid the identification of alerts and allow verification at a higher level and decision making regarding subsequent actions. In addition, it will provide clarity to all those coordinated in dengue control what and when actions need be performed and should be directly related to outbreak definition in a context specific plan. Currently, the transparency of this process is not present in outbreak response planning. This suggestion has been supported in the literature. Badurdeen et al. found that country experiences suggested that dengue outbreaks should be split into “phases” whereby tailored responses should be in place [25]. These phases include an initial response, an early response, an early response in clinical settings, and an emergency response. The algorithm in Figure 1 goes one step further in that it accounts for both alerts and related actions. It may be argued that without investment in all areas of the algorithm below, splitting a response into different levels may be aspirational.

### 4.4. Outbreak Response

Despite the evidence that outdoor fogging in national programmes has usually little impact on dengue transmission, mainly due to inadequate delivery, numerous country plans reported fogging was a key strategy [26]. It has been argued that fogging is often politically motivated, as it is highly visible and conveys the message that the government is taking action [6, 18]. One of the arguments used to justify the use of fogging is that resource poor settings face difficulties designing and implementing alternative vector-control strategies, particularly in large urban populations [12]. Analysis of the contingency plans suggests that source reduction using the community must be considered as a simple yet crucial outbreak response activity, yet community based-interventions depend solely on how engaged the community is and/or how behavioural change can be achieved [27]. It has been recommended that multifaceted interventions are more effective than single interventions, and therefore a combination of government commitment, authority involvement, and community mobilisation is best placed in order to target all possible breeding sites [28]. This needs to be clarified in future planning in order to take the focus away from fogging. 

Good clinical case management in an outbreak has been crucial in reducing dengue case fatality from 10–20% to less than 1% in some countries over the past two decades [29]. Currently, it has been found that hospital contingency measures are rarely accounted for in outbreak response planning, despite the fact that the effects of a dengue outbreak in terms of mortality and morbidity will be determined by these measures. It has been found that the key areas that need accounting for in outbreak response planning are stock management, human resources and clinical management of cases, and triage systems and space for an influx of patients [25]. Some of the suggestions from individual plans of ways to account for the strain on hospital services include an emergency dengue unit or dengue emergency room to be formed, or a triage referral system to be in place with clear communication as to how to provide adequate medical treatment in emergencies and when resources are low. It has been found that increasing surge capacity may be as simple as using mattresses on the floor, foldable beds, or more than one patient per bed [25]. However, it may be argued that these strategies may still be last resort options in crisis situations. The plans that include additional facilities in place such as opening of public places to care for patients, such as town halls or places of worship, and those that include systems as opposed to just resources to account for an influx of patients, are the most feasible and structured contingency measures. This is currently a large weakness in outbreak planning. This plan review has identified a number of different ways of obtaining staff for clinical management of dengue patients, as highlighted in the discussion above. In addition, the best ways to achieve successful training, especially in countries with weak governance or poor healthcare management, may be through hands-on training during ward rounds and case conferences [3]. The importance of emergency resources and funding for an outbreak response including clinical supplies has been highlighted as an important element of preparedness and response planning, and this must be crucial in future outbreak response planning to fill the gap that currently exists [1]. It can be recommended that all outbreak response planning should account for early responses in clinical settings, with particular focus on circulation and familiarisation of staff with the management of dengue, staff training and engagement of other authorities, and the private sector to enhance surge capacity in clinical settings [25].

### 4.5. Overall Limitations of Preparedness Planning

From the breakdown of the plans, strengths and weaknesses in dengue outbreak response planning have been identified. Despite all but one of the plans being country specific, the lack of actual context specific input may hinder the use of the plans. Many of the plans provide generic information that is deemed relatively weak in relation to implementation. Some plans clearly split the activities that shall be performed in the interepidemic period and during the outbreak, yet a frequent weakness is that there is a lack of clarity in identifying specific outbreak strategies. Some country plans advocate up scaling of preventative interventions when case numbers increase, with a number of outbreak specific procedures being neglected, such as an outbreak investigation, risk communication, health system management for hospital admissions, and human resource preparedness. 

Outbreak governance, disease surveillance, outbreak verification, and response initiation have been identified to be as crucial to an effective outbreak response as the interventions actually deployed to control the outbreak. The lack of recognition of these initial additional elements of the outbreak response collectively has led to a current lack of consistency in outbreak response planning. An additional limitation in contingency planning is the lack of communication between one stage of an outbreak and another, whereby the disciplines of surveillance, investigation, and response are separated concepts. In particular, this concerns the lack of continuity between surveillance, outbreak alerts, outbreak confirmation based on the outbreak definition, and outbreak declaration. This is compounded by the minimal documented accountability for each intervention so that plans do not define a body or person that is in charge of certain activities. 

## 5. Conclusions

The complexity of dengue dynamics and the multifaceted response that is demanded create a strong argument as to why dengue outbreak response planning requires contextual details, service structure considerations, and a capacity analysis all to be taken into account in order for success in outbreak control. Only through organic policy development will this process truly be viable. Nevertheless, this research highlights the need for a model contingency plan for dengue outbreak prediction, detection, and response as a framework to help countries develop their own more detailed plan in light of the numerous gaps and profound weaknesses identified. Particular areas to be developed in outbreak response planning are outbreak management and stakeholder collaborations, surveillance strategies that include alert thresholds for when action should be initiated, and the ‘‘who, when, how, and why” of outbreak response activities as opposed to just the “what”. In light of the lack of evidence of alert signals and surveillance thresholds identified in collaborating the country outbreak response material, it is also clear that operational research is required to test the validity of alert signals and thresholds and to identify the most cost-effective interventions that may be implemented during both an “early” response and “late” response.

## Primary Sources

 Alcadaia de Santiago de Cali Secretaria de Salud Publica Municipal (2010) *Plan de Contingencia Para la Deteccion y Control del Dengue en el Municipio de Santiago de Cali *
 Centro de Prevencion Control de Epidemias, Emergencias Y Desastres, Direccion de Salud San Martin (2011) *Plan de Contingencia Para la Prevencion Y El Control de Brote de Dengue y Dengue Grave *
 
*Dengue Control Programme in Singapore* (n.d) (No author. Source: WHO)
 
*Dengue Fever Management in Tonga*—*A Proposed Plan* (n.d) (No author. Source: WHO)
 Fiji Ministry of Health (2010) *Communicable Disease Surveillance and Outbreak Response guidelines *
 Governo de Sergipe (2011) *Plano de Contingencia Para o Controledo Dengue No Estado de Sergipe *
 
*Joint Plan of Action*: *Scaling up of Dengue Prevention and Control for the Cyclone Nargis Affected Populations* (2008) (No author. Source: WHO)
 Laos PDR (2008) *Standard Operating Procedures for Dengue *
 Ministry of Health and Quality of Life, Mauritius (2009) *Operational Plan for the Prevention and Control of Chikungunya and Dengue in the Republic of Mauritius *
 National Dengue Control Unit, Ministry of Health, Sri Lanka (2011-2015) *Strategic Plan for the Prevention and Control of Dengue Fever*/*Dengue Haemorrhagic Fever in Sri Lanka*, *2011*–*2015 *
 Papua New Guinea National Department of Health (2011) *Outbreak Manual for Papua New Guinea *
 Queensland Health (2011). *Queensland Dengue Management Plan 2010*–*2015 *
 Vietnam Red Cross Society: Hochiminh Chapter (2011) *Contingency Plan Dengue Fever Prevention and Response in Hochiminh City in 2012 *
 WHO (2011). *Comprehensive Guidelines for Prevention and Control of Dengue and Dengue Haemorrhagic Fever *
